# Clinicopathological and prognostic significance of S100A4 overexpression in colorectal cancer: a meta-analysis

**DOI:** 10.1186/1746-1596-8-181

**Published:** 2013-11-04

**Authors:** Yanqiong Liu, Weizhong Tang, Jian Wang, Li Xie, Taijie Li, Yu He, Xue Qin, Shan Li

**Affiliations:** 1 Department of Clinical Laboratory, First Affiliated Hospital of Guangxi Medical University, Nanning, Guangxi, China; 2 Department of Colorectal and Anal Surgery, First Affiliated Hospital of Guangxi Medical University, Nanning, Guangxi, China

**Keywords:** Colorectal cancer, Meta-analysis, Progression, Prognosis, S100A4

## Abstract

**Background:**

Accumulated evidence has indicated a correlation between S100A4 expression and colorectal cancer (CRC) progression. However, its prognostic significance for patients with CRC remains inconclusive. To clarify their relationship, a meta-analysis of the relevant published studies was performed.

**Method:**

PubMed, Cochrane Library, and Web of Science databases were electronically searched. All studies evaluating the prognostic value of S100A4 expression in CRC patients regarding survival and a series of clinicopathological parameters were included. The effect of S100A4 expression on the overall survival (OS) and disease-free survival (DFS) were measured by pooled hazard ratios (HRs) and 95% confidence intervals (CIs), while the effect of S100A4 expression on the clinicopathological parameters were measured by the pooled odds ratios (ORs) and their 95% CIs.

**Results:**

Eleven studies (2,824 patients in total) were included in the meta-analysis. Overall, S100A4 overexpression was significantly associated with worse OS (HR = 1.90, 95% CI: 1.58–2.29, *P* <0.001), and worse DFS (HR = 2.16, 95% CI: 1.53–3.05, *P* <0.001) in patients with CRC. Subgroup analyses showed that S100A4 overexpression was significantly correlated with poor OS in Asian, European, and Australian patients and patients treated with surgery or chemotherapy. Additionally, there were significant associations between S100A4 expression and several clinicopathological parameters (tumour location, lymph node metastasis, nodal status, TNM stage, and tumour depth).

**Conclusions:**

This meta-analysis indicates that S100A4 overexpression seems to correlate with tumour progression and poor prognosis of CRC patients. It may be a useful marker to predict progression and prognosis of CRC.

**Virtual slides:**

The virtual slide(s) for this article can be found here: http://www.diagnosticpathology.diagnomx.eu/vs/8643820431072915

## Introduction

Colorectal cancer (CRC) is one of the most frequently occurring cancers worldwide; cancer-related deaths have thus become a major public health challenge [1], being the second and third most common causes of cancer deaths in the USA and Europe, respectively [2,3]. In Asia, CRC is the fourth leading cause of mortality by cancer, and its incidence is increasing [4]. Therefore, it is clear that, despite decades of advances in its prevention and treatment, CRC remains a substantial cause of death [5]. The 5-year survival rate is approximately 85% after surgical resection performed in the early stages of CRC; however, the rate is significantly decreased (<50%) in stage III CRC with lymph node metastasis [6]. Distant metastasis (stage IV) is the most frequent cause of treatment failure and forms the highest mortality of CRC, with a 5-year survival rate of <5% [7,8]. Therefore, early detection of tumorigenesis and metastases is critical to improving treatment strategies and patient outcomes. Nevertheless, suitable predictors that can be widely used in clinical settings are not currently available and accurate diagnosis and proper monitoring of cancer patients remain important obstacles for successful cancer treatment. The development of reliable biomarkers and simple tests that are routinely applicable for early detection, progression, prognosis, and therapy monitoring is strongly needed.

S100A4, also known as metastatin (Mts1) or p9Ka [9], belongs to the S100 family that contains two calcium binding sites, including a canonical EF-hand structural motif, and is classified as a metastasis-related gene [10]. S100A4 possesses a wide range of biological functions such as regulation of angiogenesis, motility, invasion, and cell survival [10]. A large number of experimental studies have linked the S100A4 gene product to the metastatic phenotype of cancer cells [10]. Clinical evidence has also indicated a correlation between S100A4 overexpression and prognosis in several cancer types, such as bladder cancer [11], breast cancer [12], esophageal-squamous cancer [13], gastric cancer [14], lung cancer [15], and pancreatic cancer [16]. In particular, growing evidence has suggested an association between S100A4 overexpression and the clinicopathological outcomes and prognosis in CRC [17-32]. Nevertheless, inconsistent data have emerged regarding the ability of S100A4 to predict disease progression and survival in CRC. Multiple studies have shown that CRC patients with S100A4 overexpression have worse overall survival (OS) and disease-free survival (DFS) [17,21,23,24,26,27,29,32]; however, one study failed to achieve statistical significance on this association in a multivariate analysis [22].

To clarify the relationship between S100A4 expression level and its prognosis value for patients with CRC, a detailed meta-analysis of the relevant published studies was performed. To the best of our knowledge, this is the first meta-analysis showing the prognostic significance of S100A4 expression in CRC.

## Methods

This systematic review and meta-analysis was carried out in accordance with the Preferred Reporting Items for Systematic reviews and Meta-Analyses (PRISMA) statement [33].

### Search strategy and selection criteria

Studies were identified by searching PubMed, Cochrane Library, and Web of Science databases (last search updated to July 7, 2013). The following search strategy was used: “*colon cancer* OR *colon carcinoma* OR *rectum cancer* OR *rectum carcinoma* OR *colorectal cancer* OR *colorectal carcinoma*” AND “S100* OR *S100A4*”. No language restrictions were applied. To ensure that no studies were overlooked, the reference lists of relevant articles and review articles were manually searched to identify additional studies.

Studies were included if they fulfilled the following criteria: i) reporting explicit methods for the detection of S100A4 expression in CRC; ii) their endpoints were to evaluate the prognostic value of S100A4 expression in CRC patients regarding OS, DFS, and a series of clinicopathological parameters; and iii) provided a relative risk (RR) estimate (risk ratio, rate ratio) or odds ratio (OR) with the corresponding confidence interval (CI) or sufficient data to calculate them. When multiple publications on the same study population were identified or when study populations overlapped, only the most recent or complete article was included in the analysis. The comprehensive database search and study selection were carried out independently by Y. Liu and S. Li. Differences were settled by consensus involving the third author (W. Tang).

### Data extraction

Two authors (X. Qin and Y. Liu) independently extracted information using predefined data abstraction forms. Discrepancies were resolved by the third author (Y. Liu) independently extracting disputed data and consensus was reached by discussion. The following information was extracted from each included trial: i) study information (including first author’s name, year of publication, country, and sample size); ii) patient information (including age, sex, type of treatment, tumour characteristics); iii) follow-up time; iv) outcome measures: data allowing us to estimate the impact of S100A4 expression on DFS, OS, and clinicopathological parameters.

### Statistical methods

Included studies were divided into three groups for analysis: OS, DFS, and clinicopathological parameters. S100A4 was considered as having a 'high’ or 'low’ expression according to the cut-off values provided by the authors in each publication, because of variation on the definition for the 'high’ or 'low’ expression of S100A4 between studies. Hazard ratios (HRs) and their 95% CIs were combined to measure the effective value. If HRs and corresponding 95% CIs were not available, they were calculated from available numerical data using methods reported by Parmar et al. [34]. Data from the Kaplan-Meier survival curves were read using Engauge Digitizer version 4.1. Three independent persons read the curves to reduce reading variability. For the pooled analysis of the relation between S100A4 overexpression and clinicopathological parameters, ORs and their 95% CIs were combined to give the effective value. The impact of S100A4 on prognosis was considered statistically significant if the 95% CI for the overall HR did not overlap 1.

Statistical heterogeneity was assessed by visual inspection of forest plots, by performing the *χ*^2^ test (assessing the *P* value), and by calculating the *I*^*2*^ statistic [35,36]. If the *P* value was less than 0.10 and *I*^*2*^ exceeded 50%, indicating the presence of heterogeneity, a random-effects model (the DerSimonian and Laird method) was used [37]; otherwise, the fixed-effects model (the Mantel-Haenszel method) was used [38]. To investigate the possible sources of the heterogeneity, we conducted subgroup analyses based on the following three aspects: first line treatment, type of method used to obtain the HR, and study regions. A sensitivity analysis was conducted to evaluate sources of heterogeneity both in the overall pooled estimate as well as within the subgroups. In addition, potential sources of heterogeneity were investigated through graphical methods such as the Galbraith plot [39]. We assessed publication bias graphically using a funnel plot and quantitatively using the Begg rank correlation test and the Egger regression asymmetry test [40,41]. If publication bias was observed, we adjusted for the effect by the use of the Duval and Tweedie trim-and-fill method [42]. All *P* <0.05 (two-sided) were considered as significant unless otherwise specified. All analyses were performed using STATA, version 12.0 (StataCorp, College Station, TX, USA).

## Results

### Study selection and characteristics

**Figure 1:**
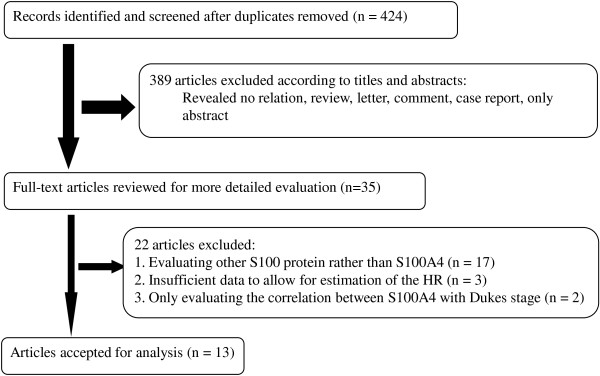
Flow chart depicting the selection of eligible studies.

The initial search yielded 424 records. After exclusion of duplicate and irrelevant studies, 13 eligible published studies were finally retrieved for the meta-analysis [17-29]. Three studies were excluded due to insufficient data to allow for estimation of the HR and OR [30-32], and two studies were excluded since they only evaluated the correlation between S100A4 with Dukes stage [43,44]. The process of article identification, inclusion, and exclusion is summarized in Figure 1 and the main characteristics are listed in Table 1.

**Table 1:** Main characteristics of all studies included in the meta-analysis

**Study**	**Country**	**Sample/Female**	**Treatment**	**Colon/rectum (n)**	**Lymph node metastasis (no/yes, n)**	**Tumor size (cm) : (n)**	**TNM stage**	**Follow-up in months**	**S100A4 assay**	**Cut-off for high expression**	**High S100A4 expression: n (%)**	**Outcome**
Gongoll, 2002 [17]	Germany	709/296	Surgery	318/391	606/103	<2: 42 2–5: 440 >5: 227	I/II: 218	NR	IHC	> 50% cancer cells stained	114 (16.1)	OS, Clinicopathological parameters
							III/IV: 491					
Boye, 2010 [21]	Norway	242/110	Mixed	163/79	185/57	NR	I/II: 165	Median 109, range 98–120	IHC	Nuclear cancer cells staining positive	73 (30.2)	OS, DFS
							III: 77					
Kwak, 2010 [22]	South Korea	127/51	Surgery	55/72	73/54	NR	I/II: 73	Median 58.7, range 1.1-101.8	IHC	20% of tumor cells stained	45 (35.4)	OS, Clinicopathological parameters
							III/IV: 54					
Wang, 2010 [23]	China	115/52	Surgery	77/38	97/18	NR	NR	Median 62, range 4-76	IHC	≥ 20% tumor cells stained	66 (57.4)	OS, Clinicopathological parameters
Huang, 2011 [24]	China	112/53	Surgery	47/65	59/53	≤5: 74; >5: 38	I/II: 57	NR	IHC	> 35% cancer cells stained	57 (50.9)	OS, Clinicopathological parameters
							III/IV: 55					
Kang, 2012 [26]	Korea	526/204	Surgery	321/205	255/271	NR	NR	Median 40.1, range 2–69	IHC	30% of tumor cells stained,	136 (25.9)	OS, Clinicopathological parameters
Kho, 2012 [27]	Australia	409/159	Mixed	451/0	NR	< 5: 205 ≥ 5: 204	I/II: 256	Median 34.6, range 0.4-351	IHC	≥ 50% cancer cells stained	45 (11.0)	OS, Clinicopathological parameters
							III/IV: 133					
Lee, 2013 [29]	Korea	333/144	Surgery	NR	240/93	NR	I/II: 187	At least 5 years	IHC	Stained cells were grade one	267 (50.0)	OS, DFS, Clinicopathological parameters
							III/IV:146					
Stein, 2011 [25]	Germany	375/205	Mixed	185/190	341/34	NR	I/II: 139	Median 24.3	RT-PCR	0.387 S100A4	143 (49.7)	DFS
							III/IV: 61			mRNA expression,% calibrator		
Cho, 2005 [18]	South Korea	124/NR	Surgery	NS	59/65	<5: 57	NR	Rang 14–38	IHC	> 30% cancer cells stained	69 (55.6)	Clinicopathological parameters
						≥ 5: 67						
Hemandas, 2006 [19]	Singapore	54/23	Mixed	34/20	46/8	<2: 5	NR	Median 65, rang 3– 104	IHC	> 20% cancer cells stained	28 (51.9)	Clinicopathological parameters
						2-5: 31 >5: 18						
Kim, 2009 [20]	Korea	73/40	Surgery	38/35	65/8	≤ 2: 4	NR	NR	IHC	> 20% cancer cells stained	40 (54.8)	Clinicopathological parameters
						2-5: 38						
						≥ 5: 31						
Giraldez, 2013 [28]	Spain	228/95	CHT	228/0	NR	NR	II: 78	Median 42,	RT-PCR	Risk score: 4.076	NR	Clinicopathological parameters
							III: 150	Range 6–152				

NR = no report; Treatment: Mixed = surgery plus chemotherapy; OS = overall survival; DFS = disease-free survival; IHC = immunochemistry; RT-PCR = reverse-transcription-polymerase chain reaction; CHT = chemotherapy.

### S100A4 expression and OS in colorectal cancer

**Figure 2:**
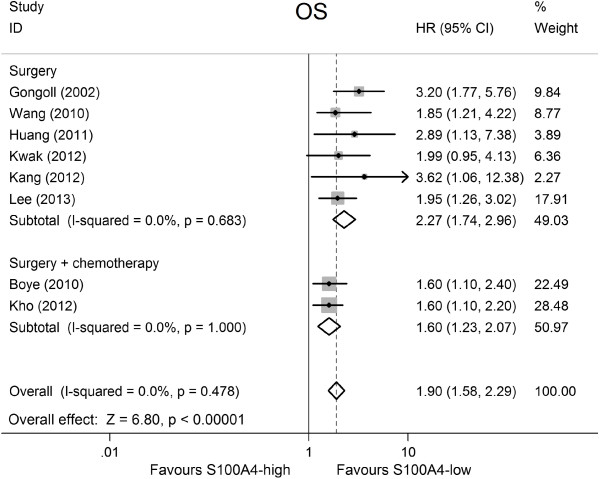
Forest plot of the association between high S100A4 expression and overall survival (OS) stratify by treatment.

Overall, eight studies including 2,615 patients reported data on S100A4 expression and OS in CRC [17,21-24,26,27,29]. Meta-analysis of the eight studies regarding the prognostic value of S100A4 expression showed that high S100A4 levels were significantly associated with poor OS (HR = 1.90, 95% CI: 1.58–2.29, *P* <0.001; Figure 2), with no heterogeneity between studies (*P* = 0.48, *I*^*2*^ = 0.0%; Table 2).

**Table 2:** Main meta-analysis results

**Analysis**	**No. of studies**	**No. of patients**	**HR (95% CI)**	***p *** **value**	**Model**	**Heterogeneity**		**Publication bias**	
						***I***^***2 ***^ ***(%)***	***p***	**Begg’s *p***	**Egger’s *p***
**Overall survival (OS)**	8	2615	1.90 (1.58–2.29)	< 0.001	F	0.0	0.48	0.06	0.03
Subgroup 1: treatment									
Surgery	6	1922	2.27 (1.74–2.96)	< 0.001	F	0.0	0.68	0.26	0.27
surgery + chemotherapy	2	693	1.60 (1.24–2.07)	< 0.001	F	0.0	1.00	-	-
Subgroup 2: type of method used to obtain the HR									
Reported HR	6	2167	1.89 (1.53–2.35)	< 0.001	F	23.3	0.26	0.13	0.06
Calculated HR	2	448	1.92 (1.34–2.74)	< 0.001	F	0.0	0.89	-	-
Subgroup 3: study regions									
Asia	5	1213	2.08 (1.55–2.80)	< 0.001	F	0.0	0.83	0.09	0.07
Europe	2	951	2.18 (1.11–4.28)	0.02	R	72.8	0.06	-	-
Australia	1	451	1.6 (1.10–2.20)	0.008	-	-	-	-	-
**Disease-free survival (DFS)**	3	950	2.16 (1.53–3.05)	< 0.001	F	0.0	0.667	1.000	0.825
**Clinicopathological parameters**			OR (95% CI)						
Age (old vs. young)	7	2040	1.24 (0.99–1.56)	0.06	F	0.0	0.91	1.00	0.87
Gender (female vs. man)	7	2113	1.15 (0.93–1.43)	0.21	F	23.2	0.25	1.00	0.81
Tumor location (rectum vs. colon)	6	1662	1.34 (1.06–1.69)	0.01	F	0.0	0.97	0.71	0.57
Differentiation (poorly vs. well and moderately)	7	1340	1.04 (0.75–1.44)	0.80	F	0.0	0.61	0.55	0.22
Lymph node metastasis (yes vs. no)	6	1671	2.62 (1.40–4.90)	0.003	R	83.7	0.00	0.26	0.07
Nodal status (N1 ~ 2 vs. N0)	4	1230	2.68 (1.57–4.55)	< 0.001	R	59.9	0.06	0.09	0.02
Distant metastasis (M1 vs. M0)	3	768	3.22 (0.65–15.84)	0.15	R	75.6	0.02	1.00	0.17
TNM stage (III/IV vs. I/II)	5	1732	3.03 (1.48–6.20)	0.002	R	82.4	0.00	0.81	0.20
Tumor depth (T 3/4 vs. T 1/2)	6	1922	1.82 (1.35–2.46)	< 0.001	F	0.0	0.49	0.71	0.99
Tumor size (size ≥ 5 cm vs. < 5 cm)	6	1523	0.88 (0.67–1.16)	0.37	F	0.0	0.48	1.00	0.99
Vascular invasion (yes vs. no)	2	977	1.29 (0.83–2.03)	0.26	F	0.0	0.48	-	-
Recurrence (yes vs. no)	2	335	2.03 (0.87–4.73)	0.100	R	68.2	0.076	-	-

HR = hazard ratio Model; OR = Odds ratio; CI = confidence interval; F = fixed-effects model; R = random-effects model.

Further subgroup analysis based on CRC patients’ treatment showed that elevated S100A4 levels were markedly related with worse OS in CRC patients treated by surgery (pooled HR = 2.27, 95% CI: 1.74–2.96, *P* <0.001), without any evidence of heterogeneity (*P* = 0.68, *I*^*2*^ = 0.0%). Moreover, high S100A4 levels were also significantly associated with lower OS in CRC patients treated by surgery plus chemotherapy (pooled HR = 1.60, 95% CI: 1.24–2.07, *P* = 0.0004; for heterogeneity: *P* = 1.00, *I*^*2*^ = 0.0%). In subgroup analysis based on type of method used to obtain the HR, the result of significance and heterogeneity remained practically unchanged. A statistically significant association was observed between S100A4 expression and the prognosis for patients with CRC among different study regions. The pooled HR was 2.08 (95% CI: 1.55–2.80, *P* <0.001) among studies from Asia, 2.18 (95% CI: 1.11–4.28, *P* = 0.02) among studies from Europe, and 1.6 (95% CI: 1.10–2.20, *P* = 0.008) among studies from Australia. Table 2 shows the main meta-analysis results.

### S100A4 expression and DFS in colorectal cancer

**Figure 3:**
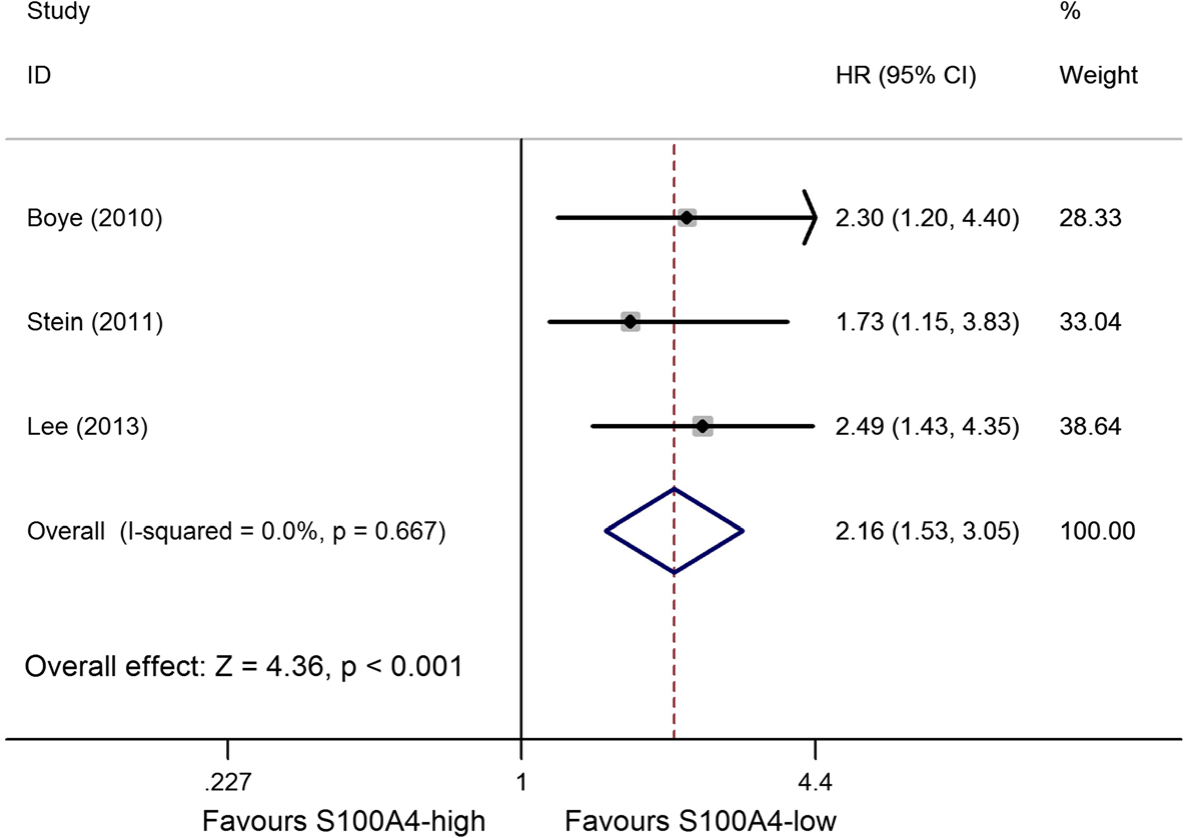
Forest plot of the association between high S100A4 expression and disease free survival (DFS).

Only three studies reported data on S100A4 expression and DFS in CRC [21,25,29]. Combined data from the three studies suggested that increased S100A4 levels were significantly correlated with DFS in CRC patients, yielding a combined HR of 2.16 (95% CI: 1.53–3.05, *P* <0.001), without significant heterogeneity in the data (*P* = 0.667, *I*^*2*^ = 0.0%) (Figure 3, Table 2). The number of studies was too small to perform a further subgroup analysis.

### S100A4 expression and clinicopathological parameters

The studies reporting data on the individual clinicopathological parameter are shown in Table 2. When the data was pooled, there were significant associations between high S100A4 expression and tumour location, lymph node metastasis, nodal status, TNM stage, and tumour depth. Specifically, the pooled ORs (95% CIs) were as follows: 1.34 (1.06–1.69) for tumour location (rectum vs. colon), 2.62 (1.40–4.90) for lymph node metastasis (yes vs. no), 2.68 (1.57–4.55) for nodal status (N1–2 vs. N0), 3.03 (1.48–6.20) for TNM stage (III/IV vs. I/II), and 1.82 (1.35–2.46) for tumour depth (T 3/4 vs. T 1/2). However, the data only suggested an evident trend towards a worse prognosis but no significant association between high S100A4 expression and CRC patients’ age (old vs. young), gender (female vs. man), differentiation (poorly vs. well and moderately), distant metastasis (M1 vs. M0), tumour size (size ≥5 cm vs. <5 cm), vascular invasion (yes vs. no), and recurrence (yes vs. no) (Table 2, Additional file 1: Figure S1).

### Sensitivity analyses

Sensitivity analyses were performed to examine whether the effect estimate was robust by sequential omission of individual studies. When a single study at a time was deleted from the above analyses, the corresponding pooled HR and OR were not significantly altered (data not shown), suggesting the robustness of the presented results.

### Publication bias

**Figure 4:**
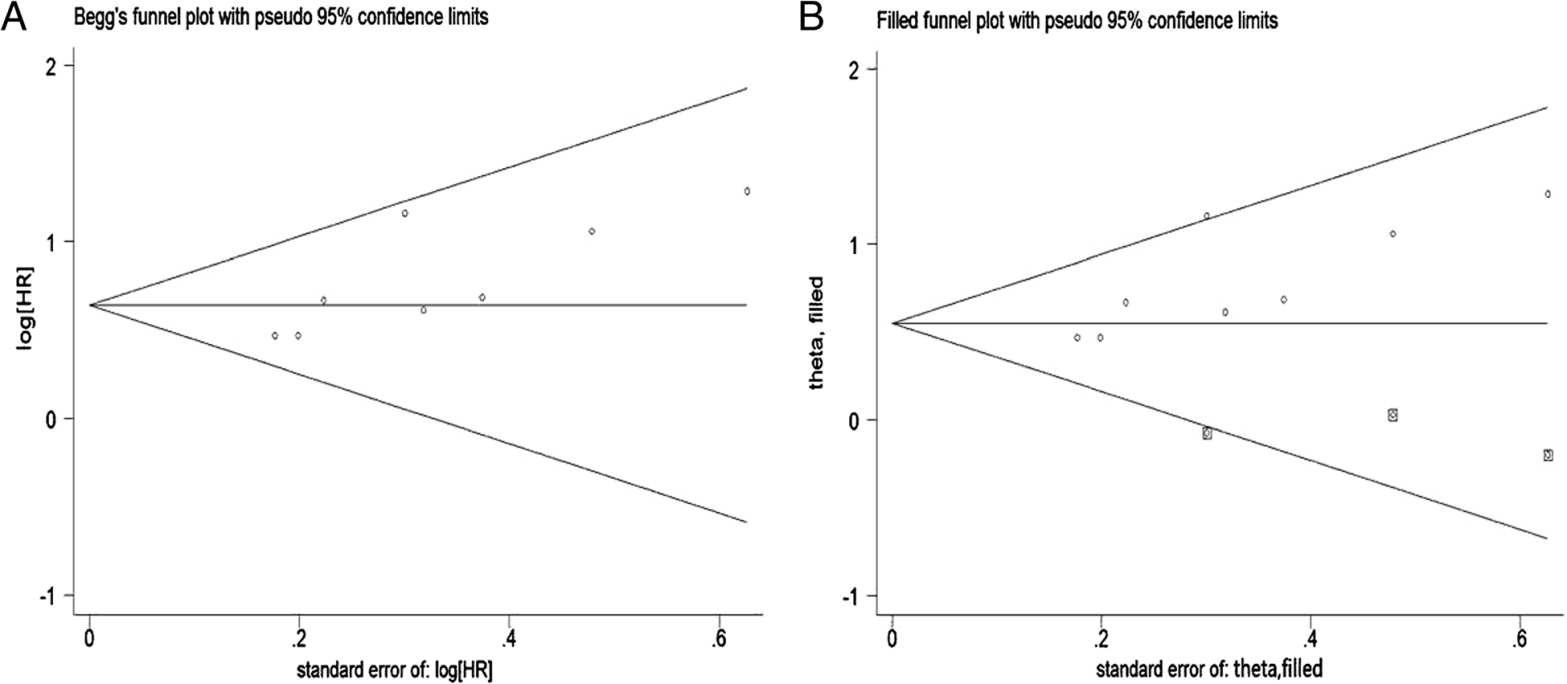
**Funnel plots of publication bias for overall survival (OS) analysis.** (**A** the original funnel plots; **B** funnel plots after trim-and-fill method adjustment).

Begg’s funnel plot and Egger’s test were performed to assess the publication bias of studies in all situations (Table 2). Publication bias was only observed in the associations between S100A4 expression and OS in patients with CRC (*P* = 0.06 for Begg’s test; *P* = 0.03 for Egger’s test) (Figure 4A). After adjustment with the trim-and-fill method (Figure 4B), the pooled association between S100A4 expression and OS in patients with CRC was also significant (fixed model: HR = 1.72, 95% CI: 1.45–2.05*, P* <0.00001; random model: HR = 1.74, 95% CI: 1.39–2.17, *P* <0.0001), and with no significant heterogeneity (*P* = 0.155), all of which indicate that the results of these meta-analyses were relatively stable and that it is unlikely that publication bias may have affected the results.

## Discussion

To date, surgical resection remains the preferred treatment strategy for CRC patients; however, not all CRC patients derive clinical benefit from such a treatment [6]. There has been special interest in identifying a novel predictive and prognostic marker to help guide clinical therapy for patients with CRC. During the past few years, many molecular markers, such as TP53 [45], KRAS, and BRAF [46], have been investigated. However, because of their limited accuracy or the lack of an adequate validation, they have not become routinely used in clinical practice. In recent years, a number of studies have been carried out to investigate the correlation of S100A4 expression and the survival and prognosis of CRC patients, although consistent results have not been reported. Therefore, we conducted a meta-analysis of the evidence obtained from all published studies in order to provide a quantitative reassessment of the association. To our knowledge, this is the first and the most comprehensive meta-analysis, to date, evaluating the association between S100A4 expression and CRC risk.

This study involves the meta-analysis of published data regarding S100A4 expression and its association with progression and prognosis in CRC. We observed a positive relationship between S100A4 overexpression and worse survival overall and among subgroups defined by treatment, type of method used to obtain the HR, and study regions. Furthermore, we also observed a significant association between high S100A4 expression and several clinicopathological parameters (lymph node metastasis, nodal status, TNM stage, and tumour depth).

Eight studies were included in the OS analysis. A European study evaluating the association S100A4 overexpression and OS was excluded because of insufficient data to estimate HR [32]. This study investigated 33 colon cancer patients who underwent colonic surgery and evaluated S100A4 expression by RT-PCR, demonstrating that colon patients with high S100A4 expression had a significantly worse OS [32]. This result was consistent with our overall analysis; it suggests that our analyses were credible enough.

S100A4 was first described in 1984 as an upregulated mRNA in a rodent model of mammary metastasis [47]. Since then, it has been reported to be involved in the pathogenicity of several diseases [48,49], and has been associated with patients’ outcome in a number of tumour types [11-16,50,51]. Importantly, all bar two [21,27] of the experimental studies examining the prognostic value of S100A4 expression published thus far, have been retrospective studies. On the other hand, most S100A4 expression studies have focused on tumours at multiple stages; there have also been several previously published reviews on this topic [10,52-54]. These reviews have shown a qualitative description about the multiple roles of S100A4 protein in tumorigenesis and metastasis and the inverted association of tumour patients’ prognosis. However, these reviews could not give a precise estimation of survival correlation. The landmark study by Gongoll et al. [17], showed that high S100A4 expression was correlated with worse prognosis in patients that received surgery. Conversely, it was concluded that S100A4 seemed to be a more valuable prognostic factor than the nodal lymph node status (pN), which lost its prognostic value in the multivariate Cox model if S100A4 was added. Subsequently, more than twelve studies investigated this topic and practically demonstrated a significant association between S100A4 overexpression and worse prognosis in CRC patients [17-29]. Our results were consistent with the previous experimental studies, and we obtained a more refined evaluation after pooling of the available evidence.

Metastasis is the main cause of death in patients with CRC. Clinicopathological parameters, such as poorly differentiated cancer, depth of wall penetration, and TNM stage, are considered the pathological risk factors for lymph node metastasis [55]. Nevertheless, these features are still insufficient to predict the existence of metastasis and are currently critically discussed, pointing to the need for new factors, either morphological or molecular, that could more precisely stratify patients into different risk categories. One candidate biomarker for the progression and prognosis of multiple malignant tumours is S100A4. Although the association of S100A4 with tumour progression has been explored in recent years, the available data have not been analysed comprehensively until now. As expected, in the present meta-analysis, the results suggested a significant association between high S100A4 expression and advanced TNM stage, nodal status, and tumour depth, as well as the presence of lymph node metastasis. Pooled data also suggested an evident trend towards higher S100A4 expression with poor differentiation, the presence of vascular invasion and distant metastases, although the statistical significance did not reach the significant level. Taken together, the pooled results in our meta-analysis support the hypothesis that S100A4 overexpression might promote CRC invasion and metastasis, and thus lead to a poor prognosis of CRC.

Obviously, the molecular mechanisms of S100A4 promotion of tumour progression need a more comprehensive understanding. Moreover, the association between S100A4 expression and worse survival should be analysed through larger multicentre prospective studies using standardized unbiased laboratory methods and well matched patients and controls. Another promising area should be the discovery of novel therapeutic strategies targeting S100A4 and suggested inhibitors, which may serve in the development of treatments for CRC metastasis.

The strengths of the present study were the rigorous search strategy, the avoidance of a language limitation, and the stringent inclusion criteria. Moreover, studies evaluating S100A4 expression through all available methods were included, which avoided the limitation of data and non-credible results. In addition, no significant heterogeneity was observed on the pooled survival analyses, which indicated that the statistical results were robust. Furthermore, the results of sensitivity analysis, subgroup analysis, and adjusting for the effect of publication bias by the trim-and-fill method did not alter and did not draw different conclusions, indicating that our results were strong. To our knowledge, this is the first meta-analysis on the association between S100A4 expression and OS, DFS and clinicopathological parameters in CRC.

The data currently available on S100A4 expression and CRC are somewhat promising, but these findings must be further confirmed by large prospective studies for the following reasons. First, studies included in the meta-analysis were mainly retrospective analyses; it is possible that other unknown confounders will bias the data. Second, the number of studies classified into pooled DFS group analysis was limited, preventing firm conclusions. Third, some publication bias was observed although significance did not alter after adjusting by the trim-and-fill method. Fourth, cut-off values of S100A4 high or low expression were different in the studies. The different cut-off value between studies may affect the results and account for the inconsistencies. However, it was difficult to provide an exact definition for 'high’ or 'low’ expression in view of the different S100A4 detection methods used. Therefore, future studies on this topic should use a consistent definition for 'high’ or 'low’ expression and use the same S100A4 detection method. Finally, subgroup analyses cannot be performed by Dukes stages due to the heterogeneity of tumour stages involved in individual studies. Given the limitations listed above, our results should be interpreted with caution.

## Conclusions

This systematic review and meta-analysis demonstrates that high S100A4 expression seemed to correlate with tumour progression and prognosis of CRC patients treated by surgery or chemotherapy in different study regions. S100A4 may be a useful marker to predict development, progression, and prognosis of colorectal cancer.

## Competing interests

The authors declare that they have no competing interests.

## Authors’ contributions

XQ and SL conceived the study idea and designed the study. YL, WT and SL reviewed the literature and performed statistical analyses. YL and XQ extracted data and drafted the manuscript. JW, LX, TL and YH reviewed and edited the manuscript. All authors read and approved the final manuscript.

## Authors’ information

Yanqiong Liu and Weizhong Tang: co-first authors.

## Supplementary Material

Additional file 1: Figure S1 Forest plot showing results of studies on the associations between S100A4 expression and clinicopathological parameters (age, gender, lymph node metastasis, tumor-node-metastasis (TNM) stage, differentiation, tumor depth, tumor size, tumor location, vascular invasion and recurrence). Click here for file
