# Supplementary material for: Clinicopathological and prognostic significance of S100A4 overexpression in colorectal cancer: a meta-analysis
Source: Diagn Pathol. 2013 Nov 4;8:181. doi: 10.1186/1746-1596-8-181 (PMC3833630; doi:10.1186/1746-1596-8-181)
Supplement: Additional file 1: Figure S1 — Forest plot showing results of studies on the associations between S100A4 expression and clinicopathological parameters (age, gender, lymph node metastasis, tumor-node-metastasis (TNM) stage, differentiation, tumor depth, tumor size, tumor location, vascular invasion and recurrence). [file 1746-1596-8-181-S1.pdf]

Supplementary Figure S1

1. Age (old vs. young);

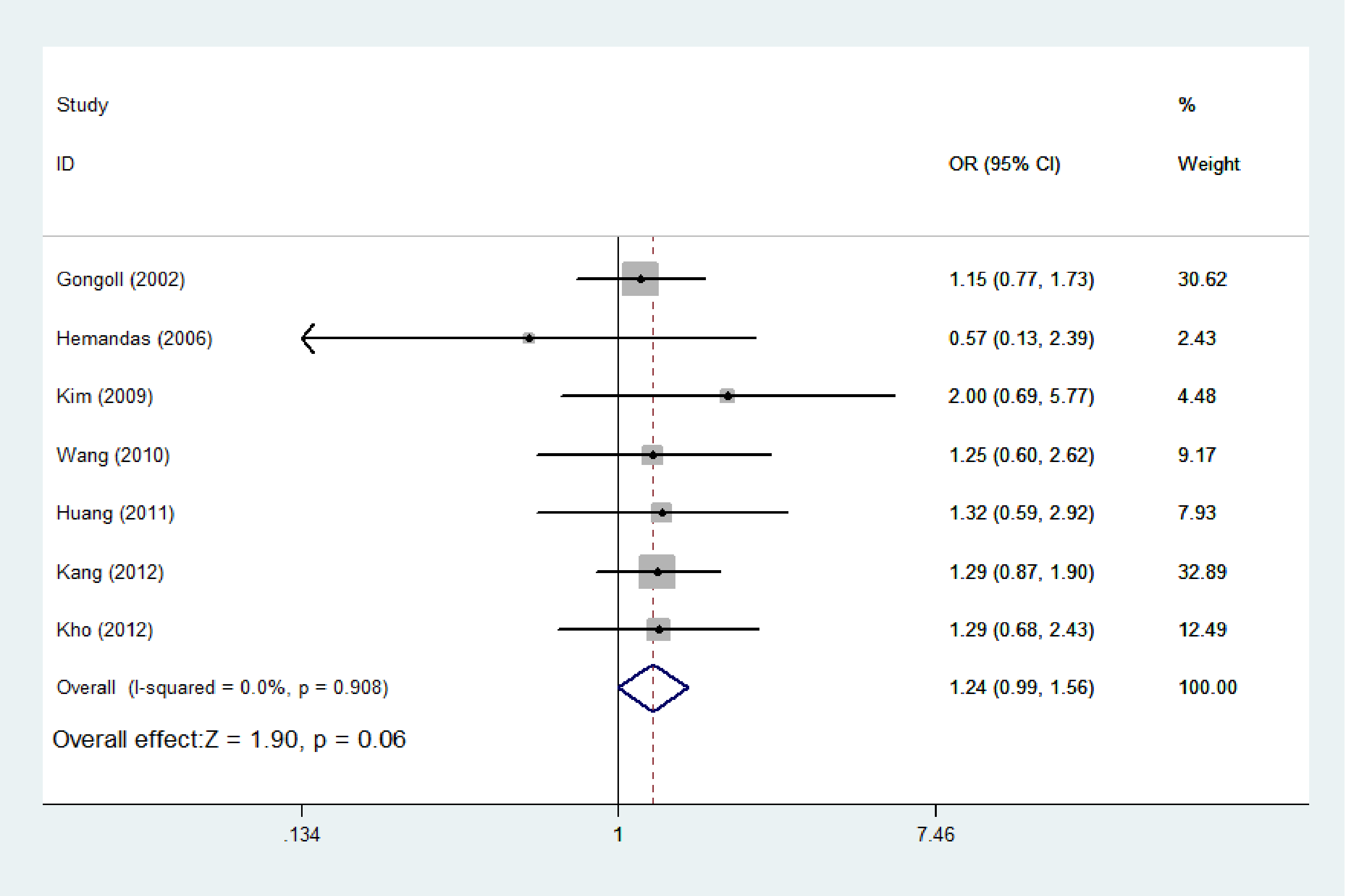

2. Gender (female vs. man):

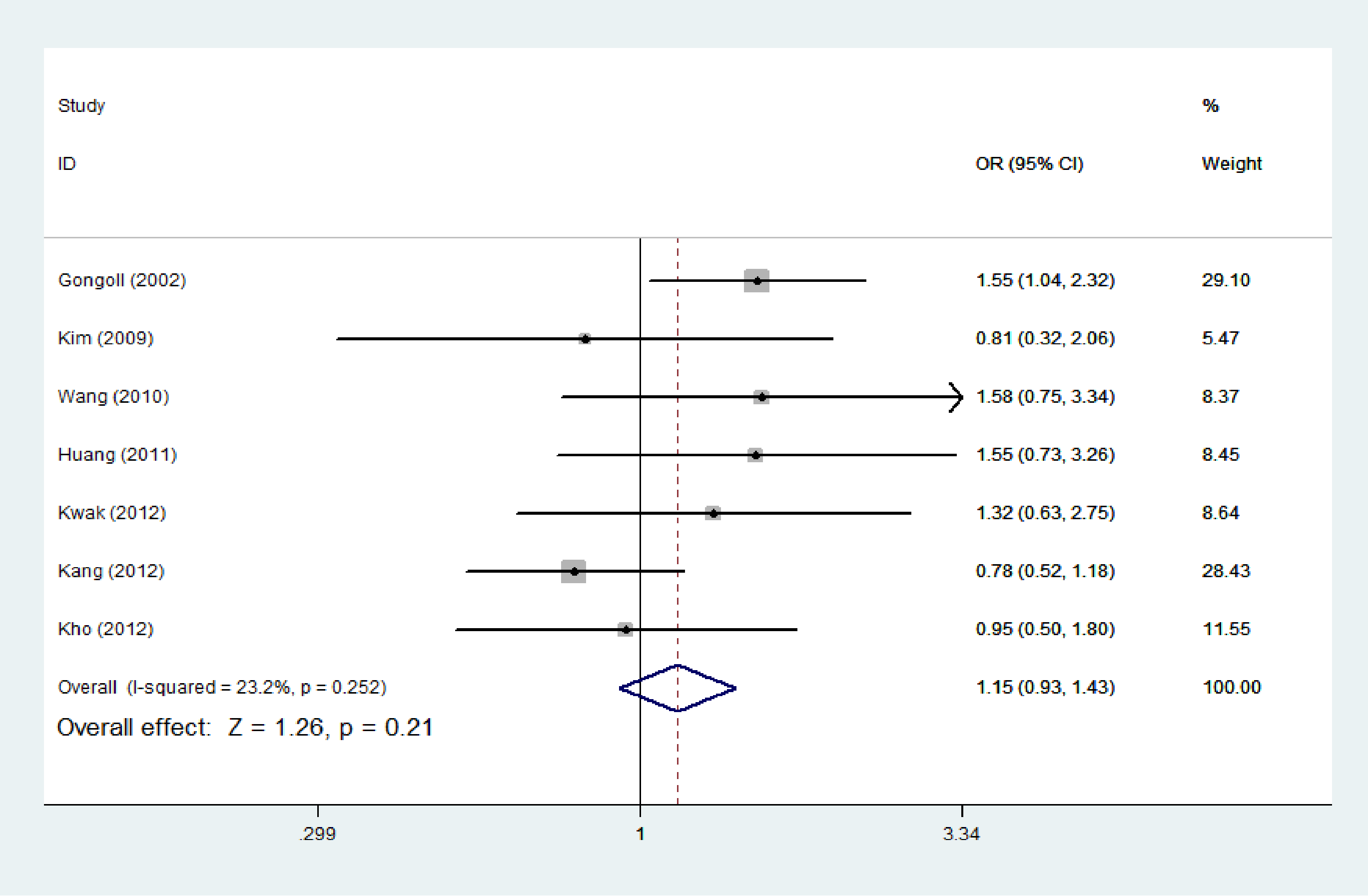

3. Tumor location (rectum vs. colon)

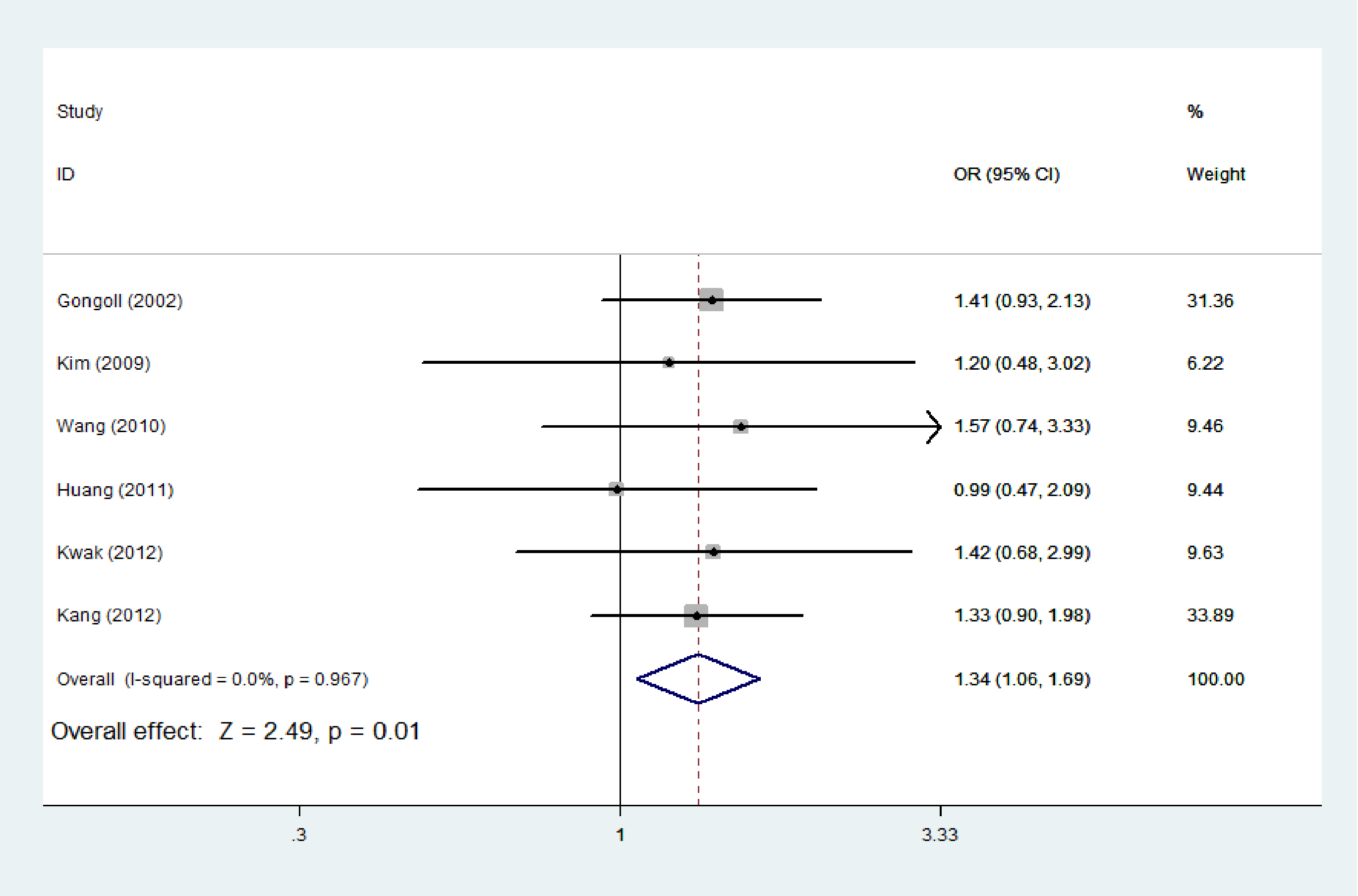

4. Differentiation (poorly vs. well and moderately)

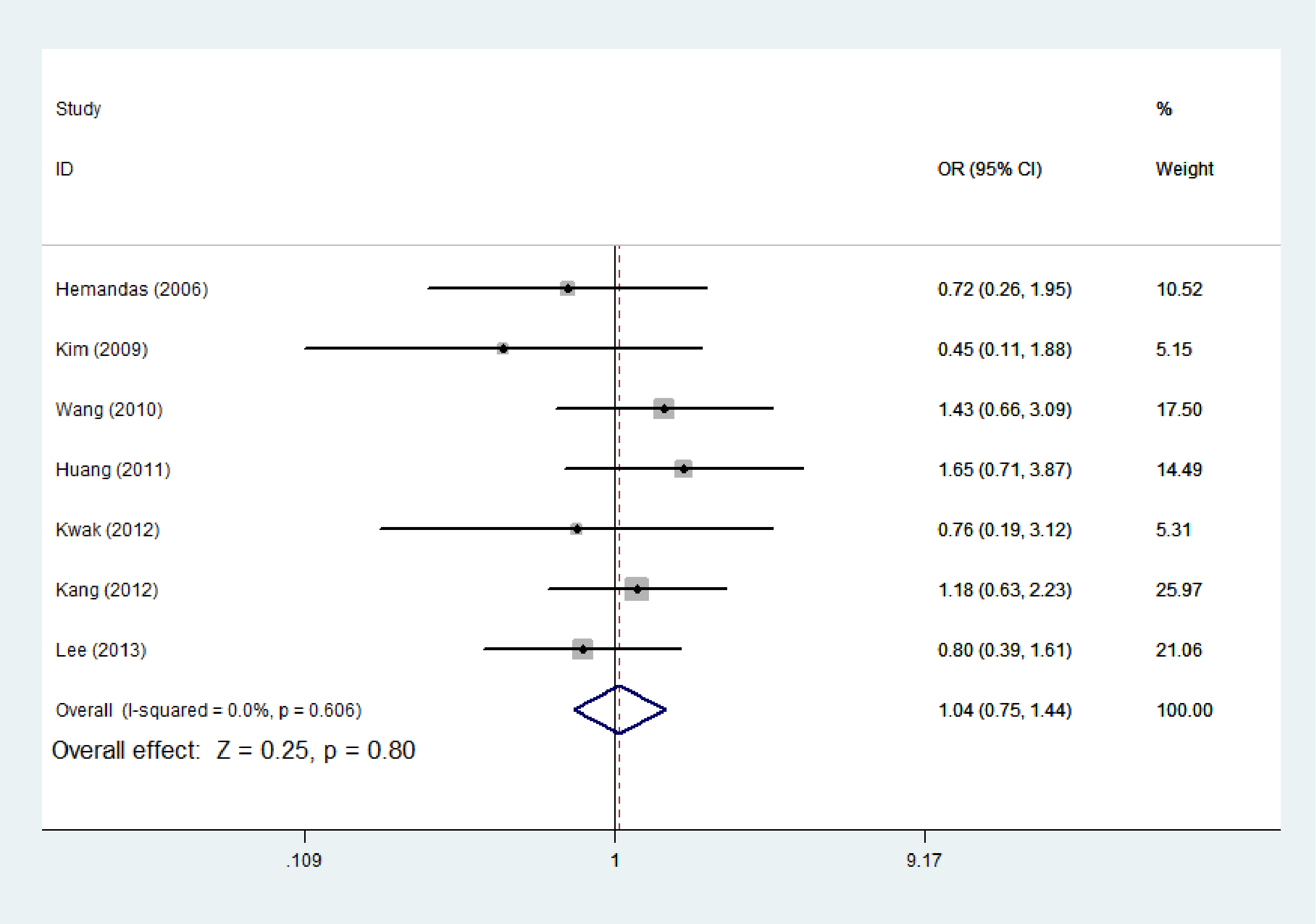

5. Lymph node metastasis (yes vs. no)

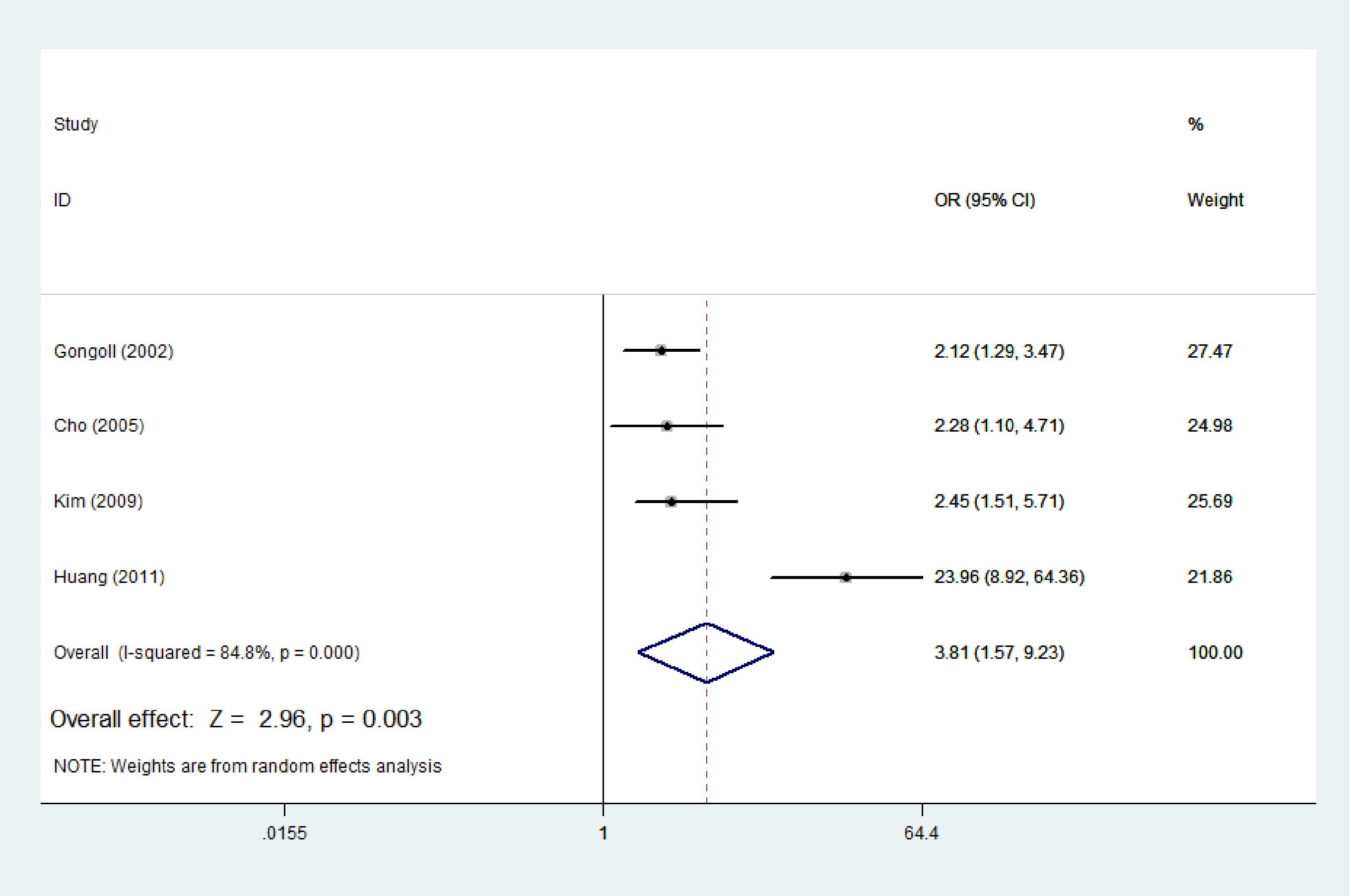

6. Nodal status(N1~2 vs N0)

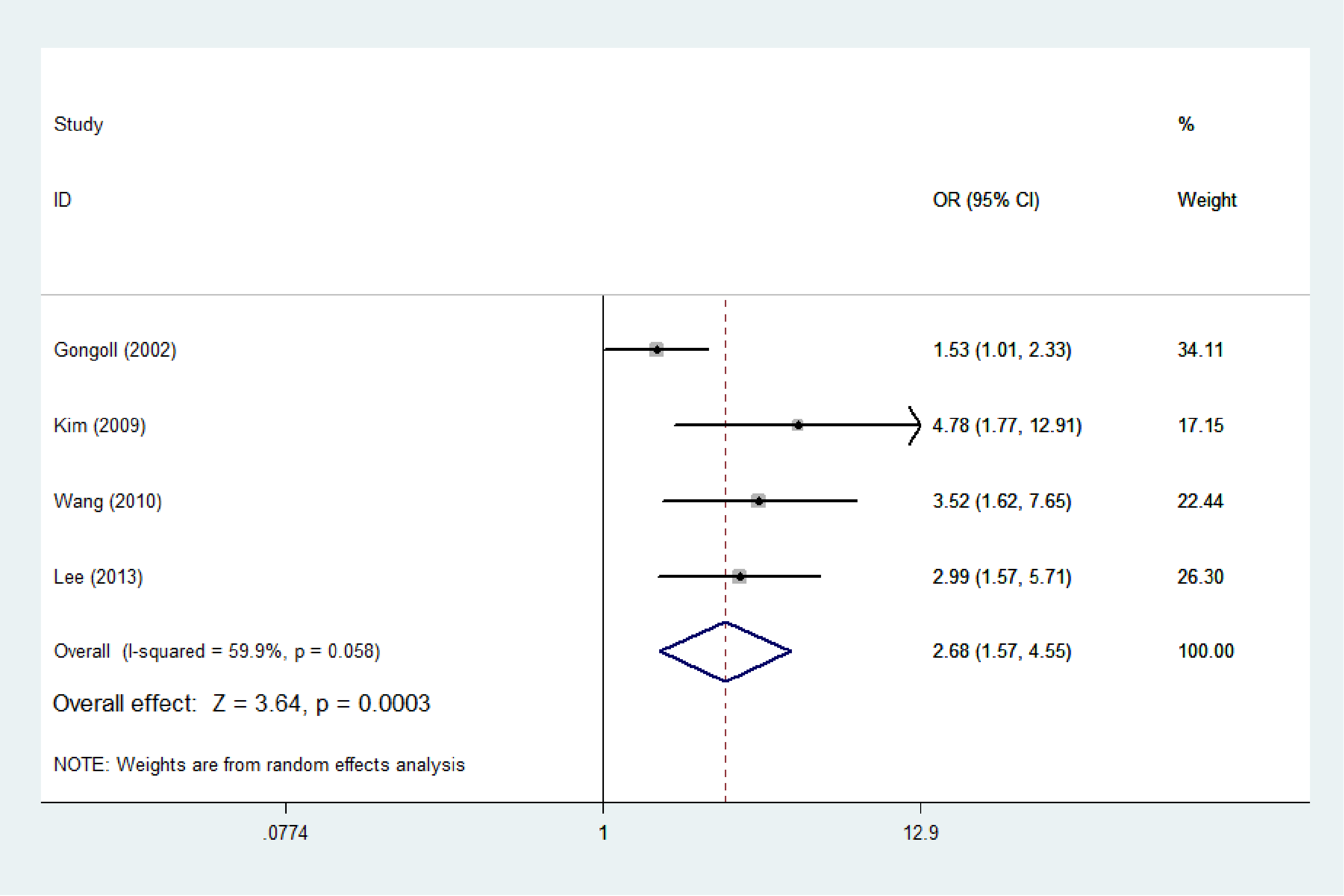

7. Distant metastasis (M1 vs. M0)

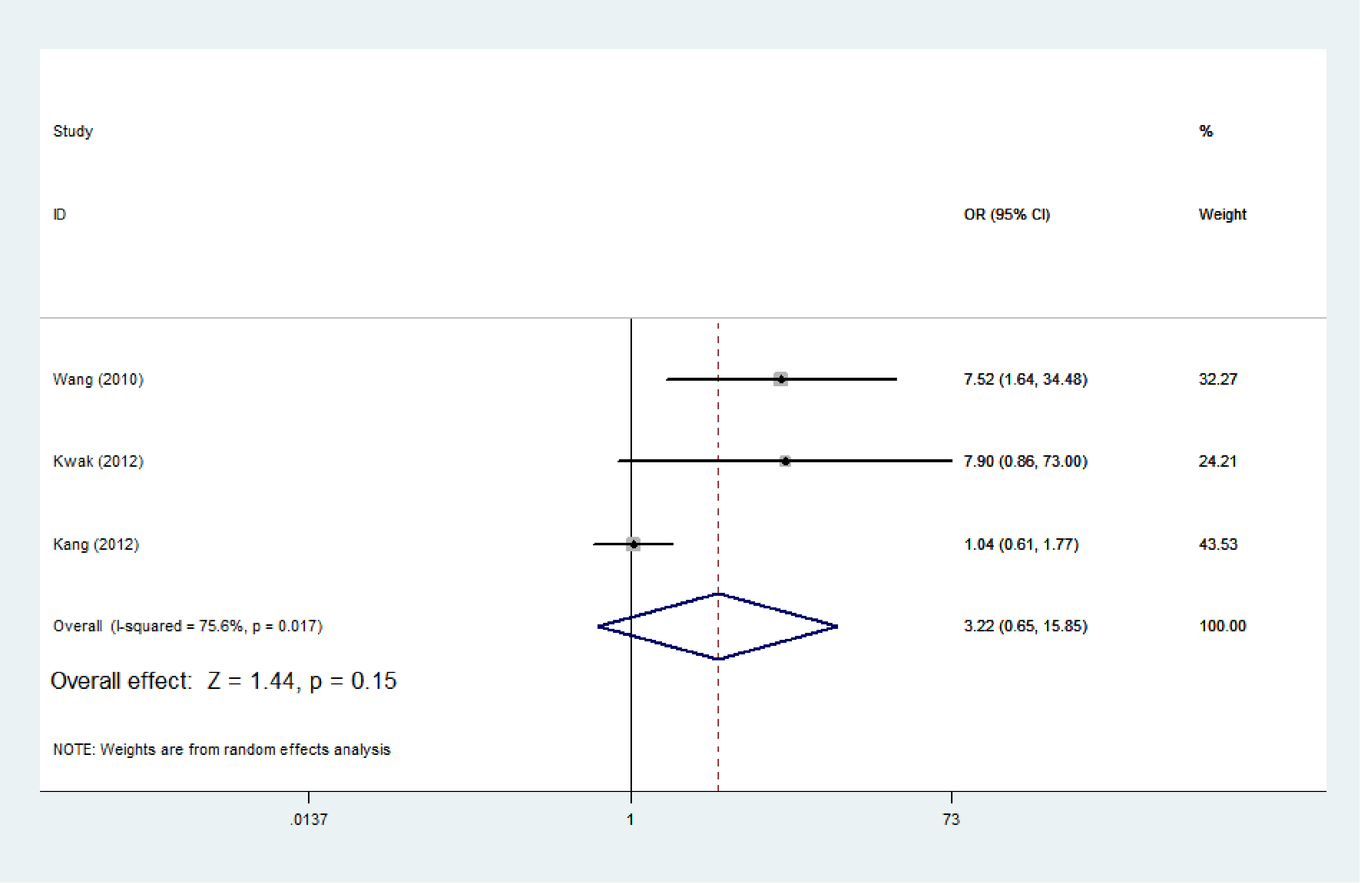

8. TNM stage (III/IV vs. I/II)

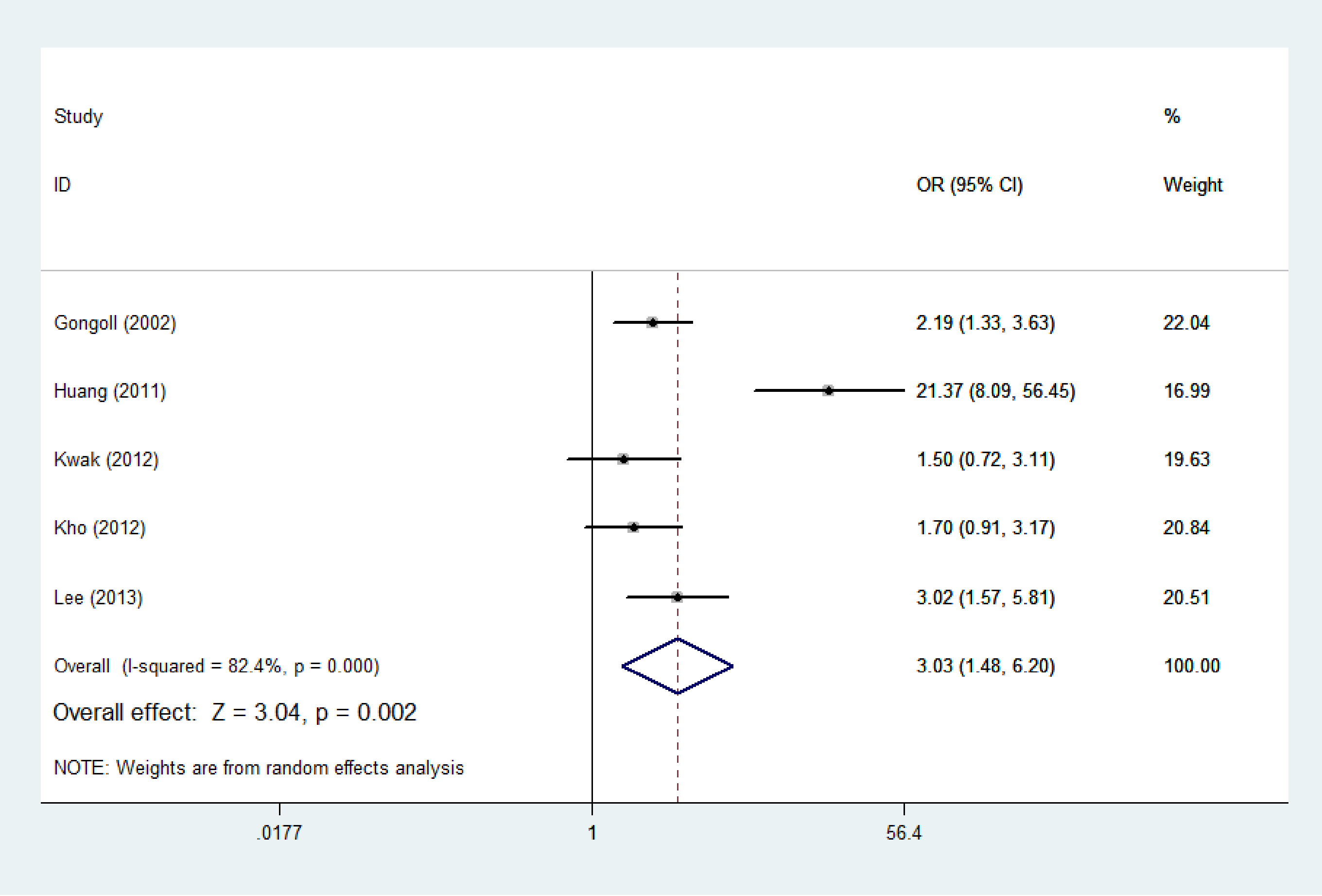

9. Tumor depth (T 3/4 vs. T 1/2)

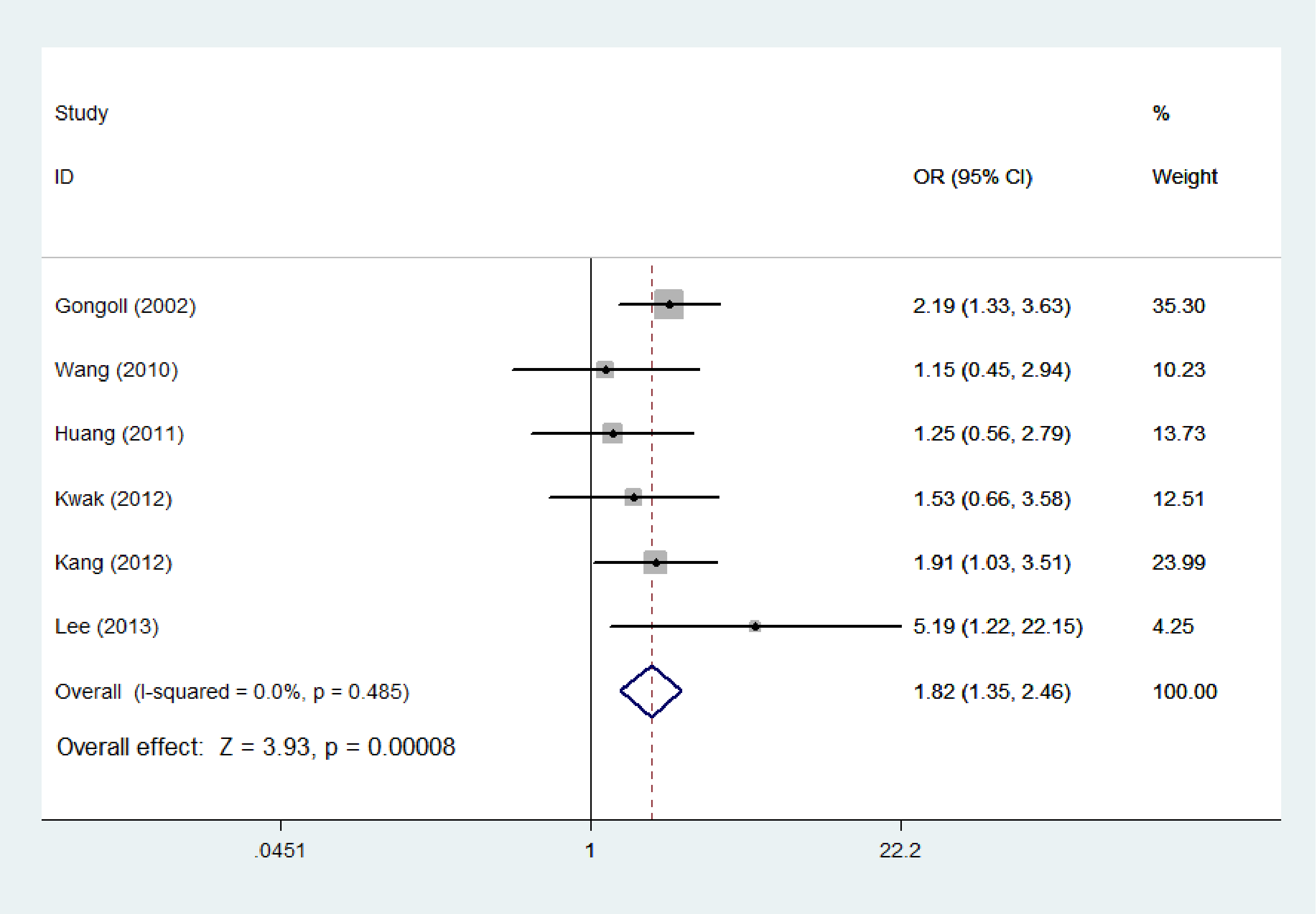

10. Tumor size (size ≥ 5 cm vs. < 5 cm)

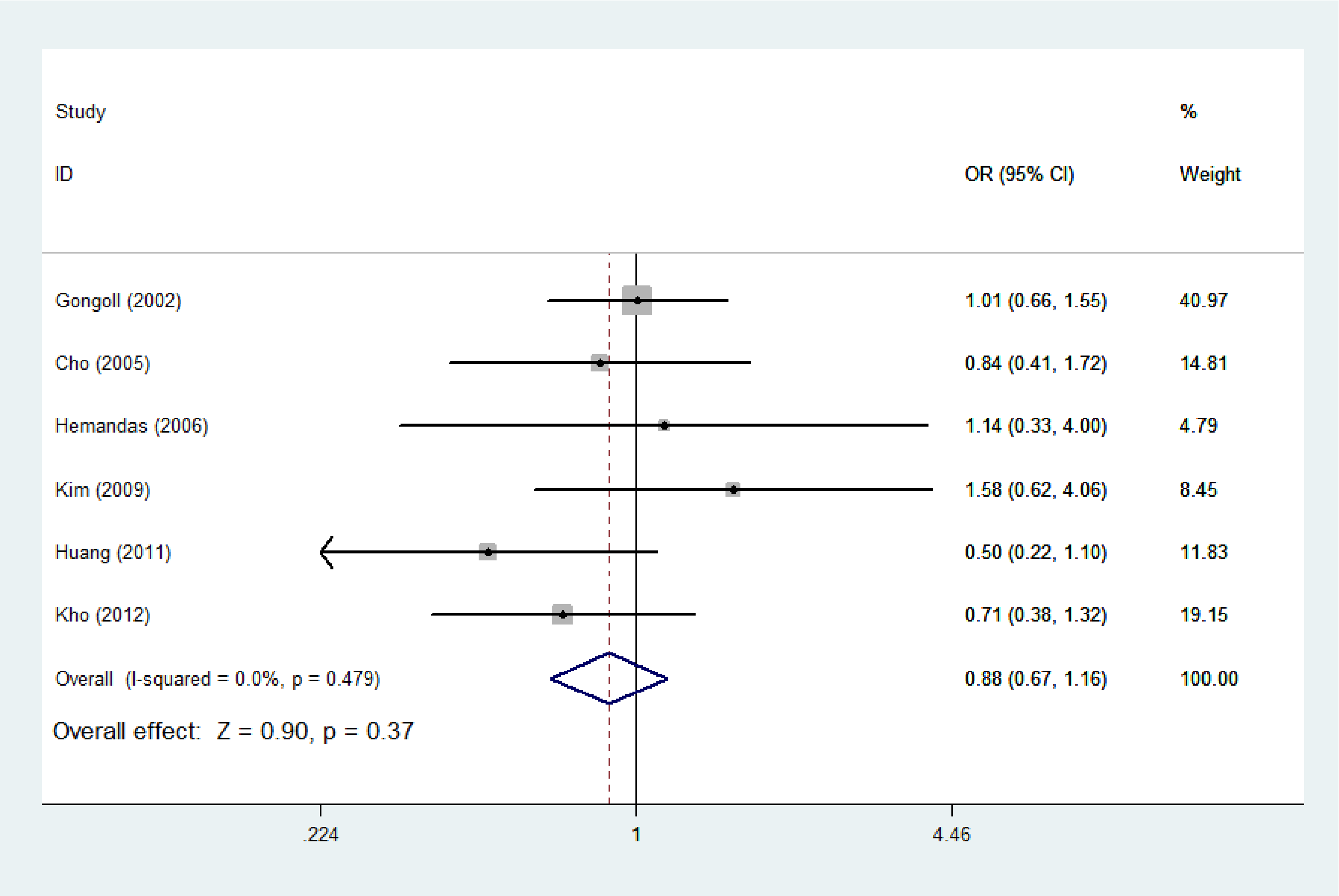

11. Vascular invasion (yes vs. no)

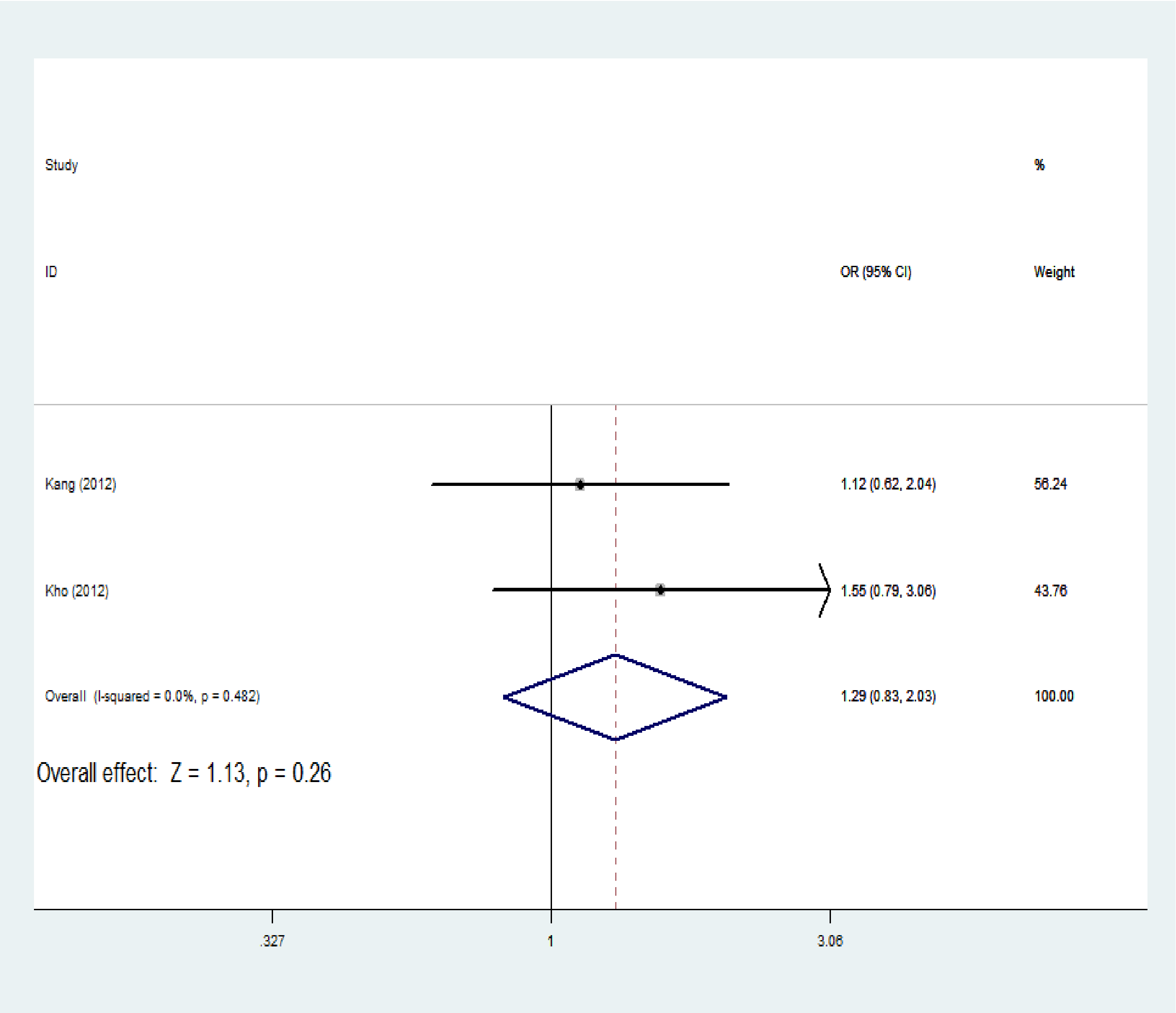

12. Recurrence (yes vs. no)

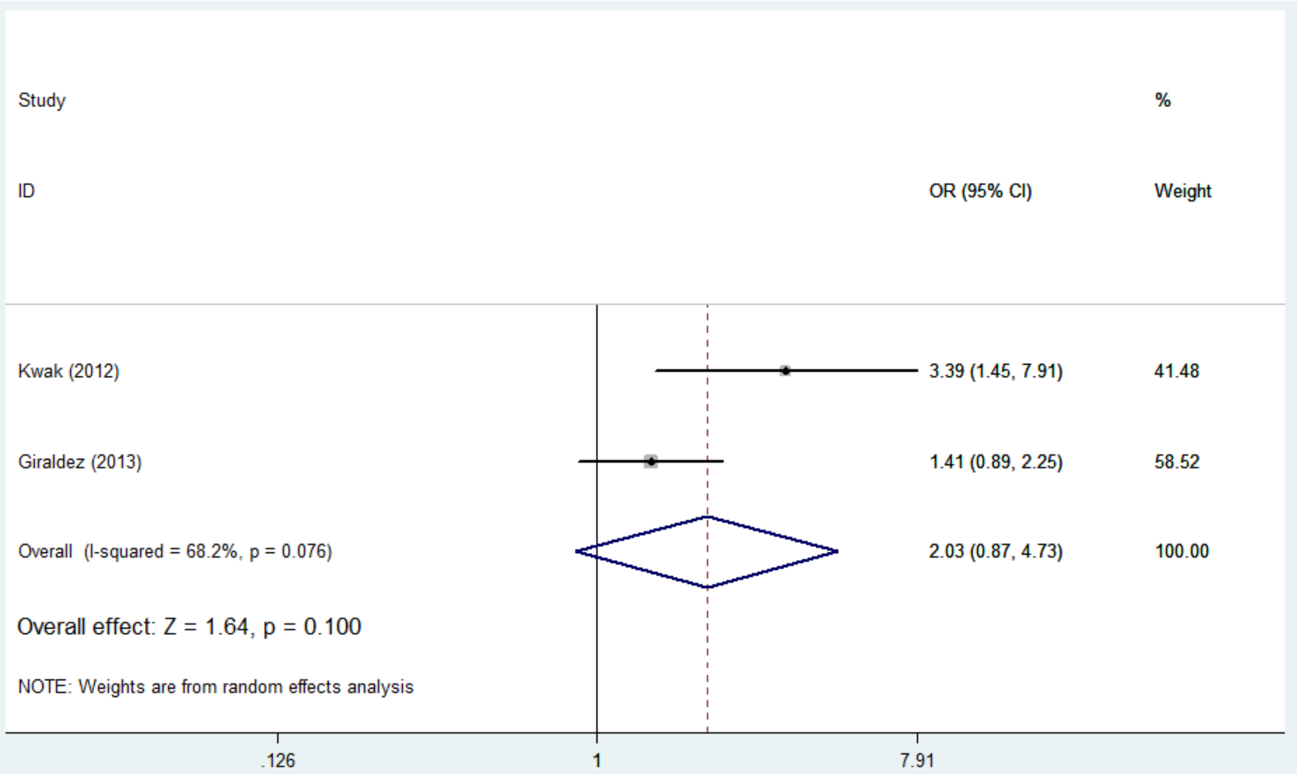

Supplementary Figure S1 Legend: Forest plot showing results of studies on the associations between S100A4 expression and clinicopathological parameters (age, gender, lymph node metastasis, tumor-node-metastasis (TNM) stage, differentiation, tumor depth, tumor size, tumor location, vascular invasion and recurrence).
